# Effect of Meteorological and Geographical Factors on the Epidemics of Hand, Foot, and Mouth Disease in Island-Type Territory, East Asia

**DOI:** 10.1155/2015/805039

**Published:** 2015-07-28

**Authors:** Chang-Chun David Lee, Jia-Hong Tang, Jing-Shiang Hwang, Mika Shigematsu, Ta-Chien Chan

**Affiliations:** ^1^Genomics Research Center, Academia Sinica, Taipei 115, Taiwan; ^2^Institute of Statistical Science, Academia Sinica, Taipei 115, Taiwan; ^3^National Institute of Infectious Diseases, Tokyo 162-8640, Japan; ^4^Research Center for Humanities and Social Sciences, Academia Sinica, Taipei 115, Taiwan

## Abstract

Hand, foot, and mouth disease (HFMD) has threatened East Asia for more than three decades and has become an important public health issue owing to its severe sequelae and mortality among children. The lack of effective treatment and vaccine for HFMD highlights the urgent need for efficiently integrated early warning surveillance systems in the region. In this study, we try to integrate the available surveillance and weather data in East Asia to elucidate possible spatiotemporal correlations and weather conditions among different areas from low to high latitude. The general additive model (GAM) was applied to understand the association between HFMD and latitude, as well as meteorological factors for islands in East Asia, namely, Japan, Taiwan, Hong Kong, and Singapore, from 2012 to 2014. The results revealed that latitude was the most important explanatory factor associated with the timing and amplitude of HFMD epidemics (*P* < 0.0001). Meteorological factors including higher dew point, lower visibility, and lower wind speed were significantly associated with the rise of epidemics (*P* < 0.01). In summary, weather conditions and geographic location could play some role in affecting HFMD epidemics. Regional integrated surveillance of HFMD in East Asia is needed for mitigating the disease risk.

## 1. Introduction

Hand, foot, and mouth disease (HFMD) is a syndrome which usually affects children below the age of five [1–3]. The causal agents are various enteroviruses, especially human enterovirus 71 (EV71) and coxsackie virus A16 (CVA16), responsible for 50% to 90% of HFMD in many episodes [1, 4, 5]. Some cases develop into severe clinical manifestations, such as encephalitis, aseptic meningitis, acute flaccid paralysis (AFP), neurogenic pulmonary edema, cardiopulmonary failure, and even death in the case of EV71 infection [6–9].

In the 1960s, both the Netherlands and USA recorded cases of EV71 [7, 10]. Afterward, severe cases with about a 10% case fatality rate occurred in Bulgaria and Hungary in 1975 and 1978, respectively [11, 12]. Meanwhile, cases with HFMD, CNS manifestations, and death were associated with EV71 in Japan in 1973 and 1978 [9, 13]. In the late 20th century, a vast death toll related to HFMD occurred in Southeast Asia. In 1997, 29 fatal cases were attributed to EV71 infection in Malaysia [14]. One year later, an outbreak of around 1.5 million estimated HFMD cases occurred in Taiwan and led to 405 severe cases and 78 dead children [1]. Since then, HFMD has become endemic in Southeast and East Asia. In particular, approximately 7.2 million probable cases of HFMD were reported in China during 2008 to 2012 [15]. To date, HFMD and enterovirus-derived severe syndromes have resulted in a heavy disease burden, especially to children, in this area.

Surveillance systems, for example, sentinel physician surveillance and national communicable disease networks, have been initiated for HFMD in these countries after the large scale outbreaks [16–18]. These surveillance systems provide timely disease information, yet HFMD still cannot be well controlled. Several studies have proposed integrated surveillance comprising disease surveillance and meteorological data to provide signals for early warning and prediction of this disease [15, 18–20]. Nevertheless, some factors have still not been closely studied. East Asia is composed of a continental land mass, peninsulas, and several island-type countries. The effects of meteorological factors on HFMD may not be universal across these areas. Thus, this study aims (1) to determine whether the known meteorological factors associated with HFMD are appropriate to island-type countries and (2) to examine whether different geographical zones and latitudinal ranges are associated with the incidence of HFMD within or between those island countries.

With a better understanding of the effects that the landform and meteorological factors may have, public health agencies will have a better system for early warning and control of HFMD and subsequent severe sequelae.

## 2. Methods

### 2.1. Data

Hand, foot, and mouth disease (HFMD) surveillance data were collected online from the National Institute of Infectious Diseases (NIID) in Japan (http://www.nih.go.jp/niid/en/survaillance-data-table-english.html), Taiwan CDC (http://nidss.cdc.gov.tw/RODS_5.aspx), Department of Health in Hong Kong (http://www.chp.gov.hk/en/sentinel/26/44/292.html), and Ministry of Health in Singapore (http://www.moh.gov.sg/content/moh_web/home/statistics/infectiousDiseasesStatistics/weekly_infectiousdiseasesbulletin.html). The studied period started from week 31 of 2012 to week 27 of 2014. The length of the studied period was 101 weeks.

**Figure 1:**
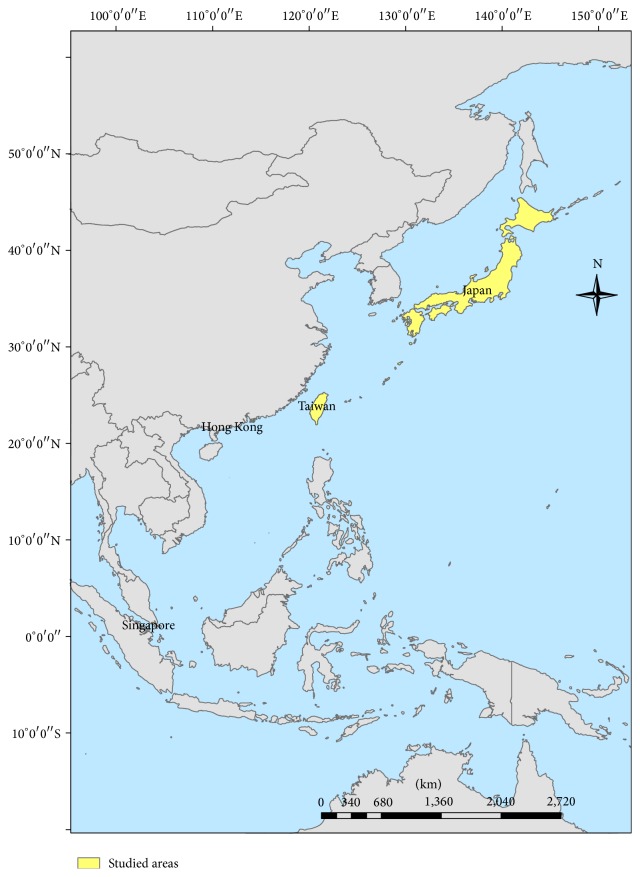
Studied areas.

**Figure 2:**
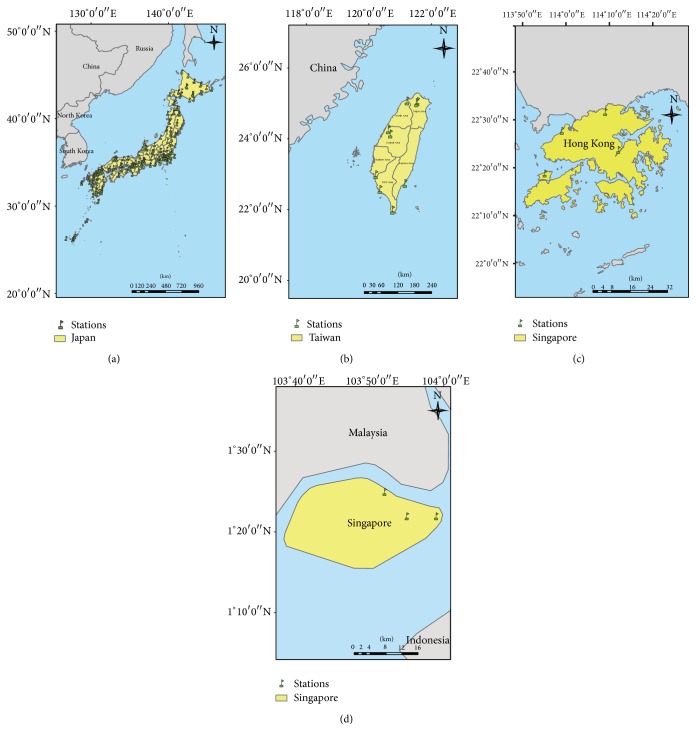
Meteorological stations in four areas in study (a) Japan; (b) Taiwan; (c) Hong Kong; and (d) Singapore.

There were 55 spatial areas including 47 prefectures in Japan, six surveillance areas in Taiwan which belonged to six control centers of Taiwan's CDC, one area in Hong Kong, and one area in Singapore. The spatial extent ranged from 1.35°N to 43.38°N in latitude and from 103.81°E to 142.57°E in longitude (Figure 1). However, the statistical units of HFMD surveillance in different countries were different. Japan used the HFMD cases per sentinel clinics and hospitals (http://idsc.nih.go.jp/iasr/20/230/de2309.html); Taiwan used the HFMD cases per 1,000 emergency room visits; and Hong Kong used two sources including general practitioners (GPs) and general practice outpatient clinics (GOPCs) from the sentinel surveillance systems [21]. In Singapore, we used HFMD incidence, which divided the HFMD cases by their population each week. The clinical definitions of HFMD are similar among the different countries and indicate a contagious enterovirus infectious disease mainly found among toddlers, with vesicles present on hands, feet, and lower limbs, inside the oral cavity (buccal mucous membrane), and/or on lips (http://www.mhlw.go.jp/bunya/kenkou/kekkaku-kansenshou11/01-05-20.html). However, the reporting mechanisms are different due to the different surveillance systems and regulations in different countries. Japan and Hong Kong's data are from sentinel hospitals and clinic surveillance. Taiwan's data are from syndromic surveillance in emergency room settings. And Singapore's data are from notifiable infectious disease surveillance since October 2000. Due to the diversity of the statistical units, we then standardized the HFMD statistics (Z rate) separately by 55 areas, which became the scale-free units for pooling analysis. The weather data were collected from the National Climatic Data Center (NCDC) of the National Oceanic and Atmospheric Administration (NOAA). The daily mean temperature (degrees Celsius, °C), mean dew point (degrees Celsius, °C), mean visibility (kilometer, km), mean wind speed (meters per second, m/s), and precipitation amount (millimeters, mm) were calculated as the average values for the corresponding weeks in each area. There were 209 weather stations included in this study after excluding the stations located at over 1,000 meters altitude and stations located on the ocean. The spatial distributions of the climate stations used are shown in Figures 2(a)–2(d).

### 2.2. Statistical Analysis

The analysis used a semiparametric generalized additive model (GAM) to examine the association between weekly standardized HFMD cases and meteorological factors [18]. We assumed a GAM with Gaussian distribution and used both cubic smoothing spline method for meteorological variables and linear term for the latitude to fit the model [22]. All GAM parameters were estimated by SAS software (SAS Institute, Cary, NC). The spatiotemporal distribution of HFMD standardized values (Z rate) was analyzed using the fishnet toolbox in ArcGIS (ArcMap, version10.2; ESRI Inc., Redlands, CA, USA).

## 3. Results

### 3.1. Temporal and Spatial Distribution of HFMD in Islands in East Asia

**Figure 3:**
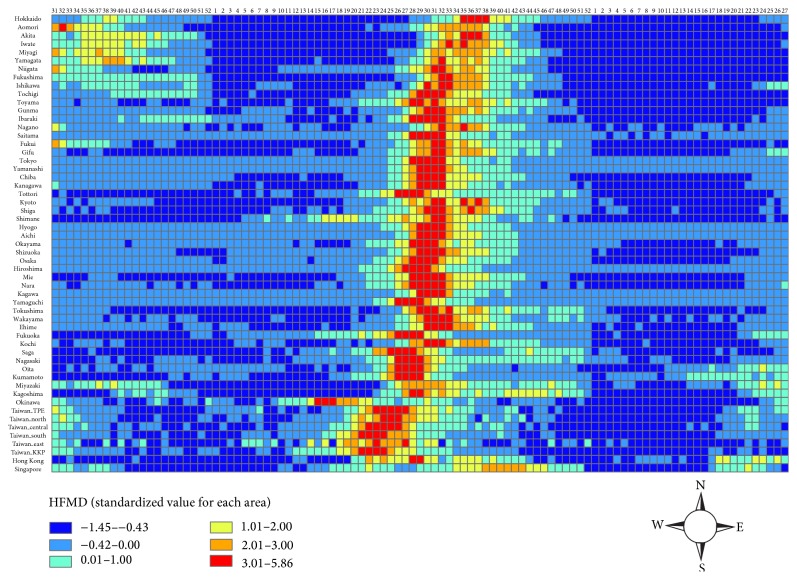
Spatiotemporal map of HFMD Z rate from week 31 of 2012 to week 27 of 2014.

All areas with HFMD Z rates among the 101 weeks are shown in the spatiotemporal map (Figure 3). Z rates in each area were standardized by all observed values during the studied period. They reflect the relative amplitude and severity of HFMD epidemic for each area. HFMD was endemic in all four areas, especially in 2013. In Japan, severe HFMD outbreaks occurred from week 25 to 38 in 2013 in almost all prefectures. Okinawa was the exception, reaching the peak earlier, from week 15 to 17 in 2013. Most of those prefectures demonstrated high Z rates of HFMD around 4 to 5 weeks long. In Taiwan, early outbreaks of HFMD showed up in southern Taiwan compared to the rest of Taiwan in 2013. The HFMD continued for at least four weeks in the three metropolitan areas (Taipei, Central Taiwan, and Kaohsiung-Pingtung (KKP)), and the highest Z rate level stopped at week 27. Compared to Japan and Taiwan, only two weeks of the highest Z rate of HFMD occurred in Hong Kong in 2013. Notably, although no Z rate was larger than 3 in Singapore, there were 6 weeks of moderate to high Z rate HFMD from week 38 to 43 in 2013. These results suggest that the epidemic timing, peaks, and durations of HFMD not only were different between countries or regions but also varied within individual areas in every country or region. Overall, the timing of the HFMD peaks was delayed with the increase in latitude.

### 3.2. Descriptive Statistics of Meteorological Factors

Five meteorological factors in those areas during the study period are summarized in Table 1. Singapore had the highest 101-week mean temperature, with standard deviation (28.3 ± 0.94°C) and mean dew point (23.8 ± 0.81°C), while those items were lowest in Japan, with 15.1 ± 8.79°C and 8.85 ± 9.28°C, respectively. Similarly, these values in Taiwan were lower than those in Hong Kong. Other measurements had no obvious order between Singapore, Hong Kong, Taiwan, and Japan. In addition, the temperature-related measurements also showed higher deviation in Japan than in other areas, which indicated the variations which occurred within Japan. Together with the decreasing trend of mean temperature from south to north, we inferred that latitude played an important role in change in temperature and would be associated with the Z rate of HFMD. Therefore, this factor was incorporated into the following GAM analysis.

### 3.3. GAM Analysis of HFMD Standardized Value

To understand the roles which latitude and meteorological factors played, the GAM analysis was performed. The final model of Z rate incorporates latitude, dew point, visibility, wind speed, mean temperature, and total precipitation. All estimates and significance levels are listed in Table 2. Based on the linear estimation of each explanatory variable from GAM, the coefficients' estimations of latitude and weekly dew point were significantly positively associated with increasing HFMD Z rate (*P* < 0.0001). The coefficients' estimations of visibility (*P* = 0.0037) and wind speed (*P* < 0.0001) were inversely associated with increasing HFMD Z rate. However, mean temperature and total precipitation were not significantly correlated with the Z rate.

**Figure 4:**
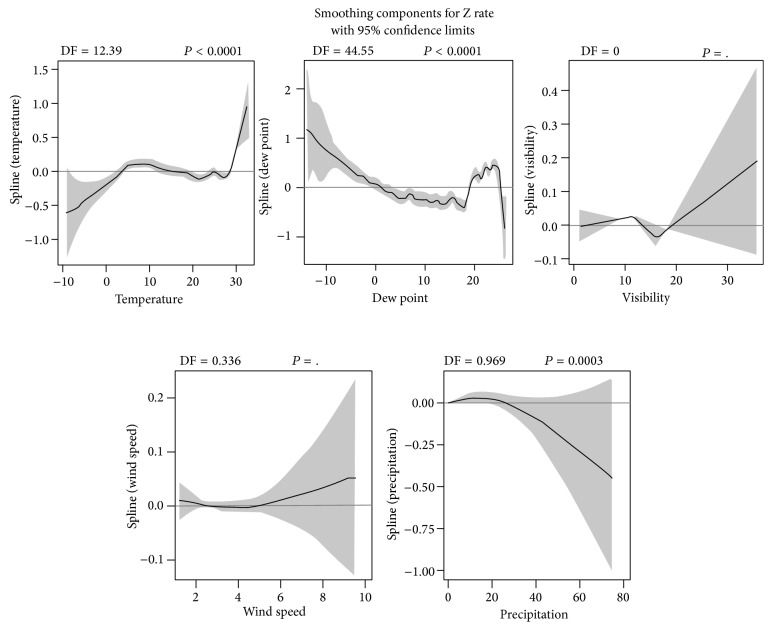
Smoothing component plots for standardized HFMD rate (Z rate).

In addition, the smoothing components plot (Figure 4) demonstrated the estimated smoothing spline functions with the linear effect subtracted out. The significant smoothers indicated that the correlations between Z rate and explanatory variables were nonlinear. The plot showed that, holding all linear and the other nonlinear terms fixed, temperature tended to increase gradually with Z rate (*P* < 0.0001), and, in addition, there was a kind of turning point for a positive effect of dew point at around of 20°C (*P* < 0.0001), with a slight positive effect overall. The Z rate tended to be lower when the total precipitation increased (*P* = 0.0003). The nonlinear effects of the visibility and the wind speed could not be estimated because their corresponding degrees of freedom (DF) were too small.

## 4. Discussion

HFMD is a common disease caused by enteroviruses that mainly attack children below the age of six in East Asia. Further clinical progress results in severe sequelae or even leads to death. Thus, HFMD is a great public health issue in this region, including mainland China (on the continent), South Korea (a peninsula), Japan, Taiwan, and Hong Kong (islands) [1, 15, 23–25]. This study incorporates meteorological data, HFMD data, and spatiotemporal analysis to reshape the epidemiological features of this disease occurring in islands during the study period. Dynamic changes and duration of HFMD severity among different parts of study sites were demonstrated clearly through the integrated surveillance. For those four epidemic islands, including Japan, Taiwan, Hong Kong, and Singapore in this study, dew point was a key meteorological factor associated with HFMD. Nevertheless, latitude difference plays an important role that even surpasses the impact of dew point on HFMD. Why this factor affects the disease rate so much is still an enigma. However, East Asia is more than 3,500 km from south to north and covers tropical, subtropical, and temperate climate zones, which are defined by climate factors and latitude. This results in different climate features in the different climate zones, including temperature variations and ranges, directions of monsoon in different seasons among climate zones, and rainfall. Those phenomena bring direct effects to the islands in East Asia; for example, typhoons strike Japan, Taiwan, and Hong Kong in summer and autumn with high-intensity rainfall that causes floods and disease outbreaks [4, 26]. Of note, HFMD is not an exception to the rule that the relative risk of enterovirus infection will increase after extreme precipitation in Taiwan [19].

In contrast, mainland China represents the continental landform, which can be divided into several regions according to both meteorological factors and latitude. In a long-term population-based study of HFMD in China, increasing amplitude of HFMD outbreaks was shown to accompany the increase of latitude in southern China [15]. Studies also mentioned that seasonal variations in China of HFMD were associated with precipitation, sunshine, temperature, and air pressure, consistent with other studies in China [18, 20, 27, 28]. In our study, only higher dew point (a predictor for humidity) was found to be positively associated with increasing HFMD Z rate. This factor is in accord with the previous findings from other studies [15, 28]. Although temperature overall did not show a linear positive effect on HFMD Z rate, the results in the smoothing components showed that temperature above 28°C had a persistent positive effect on Z rate and temperature below 5°C had a negative effect on Z rate. These findings might result from the environmental niche condition of HFMD epidemic being during the hot season. However, findings for precipitation differed between our study and previous ones [15, 19, 28]. A possible reason is that, for our study site, the mean total precipitation only differed by 0.04 inches (0.16 to 0.20) or 1.0 mm. This tiny difference would not affect the Z rate among the island type landforms in East Asia. From the GAM result, low visibility and low wind speed were significantly related to the elevation of Z rate of HFMD. Although low wind speed was also found in a transmission modeling study, there were no direct mechanisms for these negatively associated factors. A possible implication would be that higher wind speed creates good airflow, which may shorten how long the virus particles remain somewhere. Visibility was highly correlated with sunshine, which was inversely correlated with HFMD [29].

This study has some limitations. First, HFMD is caused by several enteroviruses. In this study, there was no detailed information on causal agents, for example, EV71 or CVA16 are responsible for the outbreaks among islands during the study period. This may affect the disease duration or peak of outbreaks. Second, the transmission chain of HFMD among areas defined in this study was not taken into consideration. This also impacts the spread of HFMD between or within the study areas. Finally, the studied period was too short to make strong conclusions, and extended data are needed for validating the association found in this study.

## 5. Conclusion

In conclusion, we found out that latitude is a major risk factor for increasing the Z rate of HFMD in islands in East Asia. In addition, only some meteorological factors (dew point, low visibility, and low wind speed) were found in previous mainland China studies to be associated with the outbreaks. This indicates that island-type geographical areas have intrinsic differences from continental areas, both in climate impact and in causes of HFMD. Since HFMD strikes East Asia every year and there are no effective pharmaceutical treatments for this disease, integrated surveillance with timely multiple surveillance sources and meteorological information in different countries can assist in the control and mitigation of the impacts of HFMD in East Asia. Also, these results can be applied to islands in other oceans for predicting HFMD or other infectious diseases.

## Figures and Tables

**Table 1:** Mean, standard deviation, coefficient of variation, and quantiles of meteorological factors among four regions in study.

Factors	Regions (size, *n*)	Japan (*n* = 47)	Taiwan (*n* = 6)	Hong Kong (*n* = 1)	Singapore (*n* = 1)
Temperature (°C)	Mean ± SD	15.1 ± 8.79	23.9 ± 4.47	23.0 ± 5.28	28.3 ± 0.94
	CV (%)	58.2	18.7	23.0	3.31
	*Q*1, *Q*3	7.27, 22.7	20.1, 28.0	18.7, 27.7	27.6, 28.9

Dew point (°C)	Mean ± SD	8.85 ± 9.28	18.7 ± 4.73	18.1 ± 6.23	23.8 ± 0.81
	CV (%)	104.8	25.3	34.4	3.40
	*Q*1, *Q*3	0.64, 17.6	14.8, 23.2	14.4, 24.0	23.6, 24.3

Visibility (km)	Mean ± SD	14.3 ± 4.90	8.97 ± 5.02	7.06 ± 3.67	7.07 ± 3.59
	CV (%)	34.1	56.0	52.0	50.8
	*Q*1, *Q*3	12.6, 16.8	5.32, 12.1	1.96, 9.74	1.88, 9.59

Wind speed (m/s)	Mean ± SD	3.05 ± 0.86	3.23 ± 1.58	3.14 ± 0.51	2.50 ± 0.86
	CV (%)	28.1	49.0	16.2	34.5
	*Q*1, *Q*3	2.45, 3.53	2.11, 3.80	2.79, 3.39	1.90, 2.75

Total precipitation (mm)	Mean ± SD	3.99 ± 5.06	4.11 ± 8.64	5.20 ± 8.40	4.20 ± 4.88
	CV (%)	126.9	210.4	161.6	116.2
	*Q*1, *Q*3	0.80, 5.06	0.00, 4.04	0.00, 6.35	0.76, 5.73

CV indicates coefficient of variation (standard deviation/mean ∗ 100%).

**Table 2:** Parameter estimates of linear terms in general additive model (GAM).

Variable	Estimate	Standard error	*P* value
Latitude	0.0465	0.0020	<0.0001^*^
Mean temperature	0.0031	0.0063	0.5917
Dew point	0.0566	0.0060	<0.0001^*^
Visibility	−0.0066	0.0023	0.0037^*^
Wind speed	−0.0514	0.0118	<0.0001^*^
Total precipitation	0.0018	0.0021	0.4100

^*^ *P* value < 0.05.
